# CD44 is a direct target of miR-199a-3p and contributes to aggressive progression in osteosarcoma

**DOI:** 10.1038/srep11365

**Published:** 2015-06-16

**Authors:** Yan Gao, Yong Feng, Jacson K. Shen, Min Lin, Edwin Choy, Gregory M. Cote, David C. Harmon, Henry J. Mankin, Francis J. Hornicek, Zhenfeng Duan

**Affiliations:** 1 Sarcoma Biology Laboratory, Center for Sarcoma and Connective Tissue Oncology, Massachusetts General Hospital and Harvard Medical School, Boston, MA 02114; 2 Department of Clinical Laboratory, The Third Affiliated Hospital of Zhengzhou University, Zhengzhou 450052, Henan Province, China

## Abstract

Osteosarcoma is the most common primary bone malignancy in children and adolescents. Herein, we investigated the role of cluster of differentiation 44 (CD44), a cell-surface glycoprotein involved in cell-cell interactions, cell adhesion, and migration in osteosarcoma. We constructed a human osteosarcoma tissue microarray with 114 patient tumor specimens, including tumor tissues from primary, metastatic, and recurrent stages, and determined the expression of CD44 by immunohistochemistry. Results showed that CD44 was overexpressed in metastatic and recurrent osteosarcoma as compared with primary tumors. Higher expression of CD44 was found in both patients with shorter survival and patients who exhibited unfavorable response to chemotherapy before surgical resection. Additionally, the 3′-untranslated region of CD44 mRNA was the direct target of microRNA-199a-3p (miR-199a-3p). Overexpression of miR-199a-3p significantly inhibited CD44 expression in osteosarcoma cells. miR-199a-3p is one of the most dramatically decreased miRs in osteosarcoma cells and tumor tissues as compared with normal osteoblast cells. Transfection of miR-199a-3p significantly increased the drug sensitivity through down-regulation of CD44 in osteosarcoma cells. Taken together, these results suggest that the CD44-miR-199a-3p axis plays an important role in the development of metastasis, recurrence, and drug resistance of osteosarcoma. Developing strategies to target CD44 may improve the clinical outcome of osteosarcoma.

Osteosarcoma is an aggressive primary sarcoma of bone, and is the eighth-most common form of childhood malignancy^1^,^2^. The 5-year survival for patients with localized osteosarcoma remains at 60–70% with multi-agent chemotherapy treatment together with surgical techniques^3^. Up to 20–25% of patients may have detectable metastatic disease at the time of diagnosis^4^. Metastatic osteosarcoma usually has unsatisfactory rates of response to the current standard chemotherapy, resulting in poor prognosis. The 5-year survival rate for patients with aggressive metastases is only 10–30%^5^. So far, the mechanisms controlling the aggressiveness of osteosarcoma have not been clearly characterized. The cellular and molecular mechanisms underlying metastasis formation and the development of chemoresistance in osteosarcoma remain unclear^6^.

High expression of some specific cell surface proteins has been found to contribute to tumor initiation, progression, and poor clinical outcome. Among these proteins, cluster of differentiation 44 (CD44) is a receptor for hyaluronic acid (HA) and can interact with other ligands, such as osteopontin, collagens, and matrix metalloproteinases (MMPs). The binding of different ligands to CD44 triggers direct cross-signaling between different signaling pathways, including HER2, Src kinase, and ERK^7^. Overexpression of CD44 enhances tumor cell growth, cancer stem cell differentiation, chemoresistance, and metastases^7^,^8^. Mice with disruption of the CD44 gene showed virtually ablated metastasis formation in osteosarcoma^9^. The CD44-HA interaction has been found to enhance lung metastasis and chemoresistance in osteosarcoma cells^10^. Lung metastasis of osteosarcoma can be accelerated by upregulation of CD44 *in vivo* in mouse models^11^. However, few reports have studied the role of CD44 in a larger number of osteosarcoma patients with well-established clinical information.

MicroRNAs (miRNAs, miRs) play critical roles in the regulation of genes involved in controlling tumor pathogenesis and progression. miRs bind to the 3′-untranslated region (3′-UTR) of specific gene mRNAs and participate in the regulation of many biological functions in tumor cells^12^. Several reports also demonstrated that CD44 is the direct target of miR-34 and miR-328^13^,^14^. In addition, we have previously identified miR-199a-3p as a significantly decreased miR in osteosarcoma^15^. In the current study, we evaluated the role of CD44 in osteosarcoma progression and also identified that miR-199a-3p could modulate the drug sensitivity of osteosarcoma through targeting CD44.

## Results

### CD44 is overexpressed in osteosarcoma cell lines and metastatic/recurrent osteosarcoma tissues

**Figure 1:**
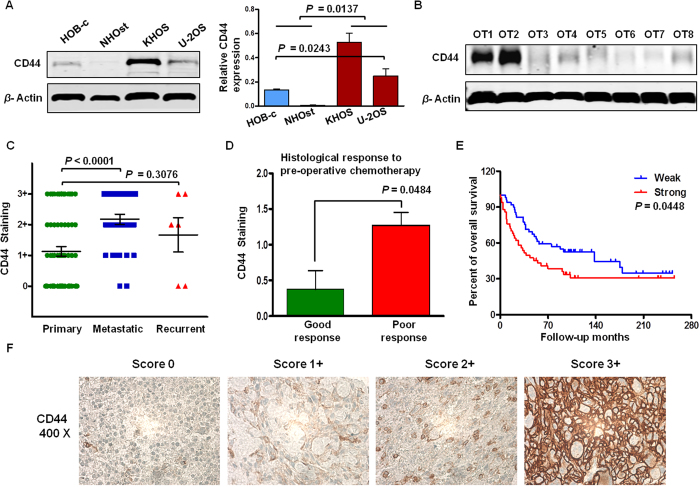
Expression of CD44 in osteosarcoma tissues, osteosarcoma/osteoblast cell lines, and its clinical significance. **A** expression levels of CD44 in osteoblast and osteosarcoma cell lines were assessed by western blot. **B** expression levels of CD44 in eight osteosarcoma tissue specimens was determined by western blot. **C** distribution of CD44 immunohistochemical staining scores in primary, metastatic, and recurrent osteosarcoma tissues. **D** comparison of CD44 immunostaining density between good response and poor response osteosarcoma tissues based on the histological necrosis percentage after pre-operation chemotherapy. E correlation between expression of CD44 (CD44 staining ≤ 1+ and CD44 staining ≥ 2+) and overall survival in osteosarcoma patients. **F** representative pictures of CD44 expression in osteosarcoma tissues.

We examined the relative protein levels of CD44 in osteoblast and osteosarcoma cell lines. There was modest expression of CD44 in HOB-c and minimal expression of CD44 in NHOst osteoblast cell lines. However, KHOS and U-2OS exhibited significantly higher expression of CD44 (*P* = 0.0137, Fig. 1A). To confirm these cell lines data in primary tumor tissues, eight primary osteosarcoma specimens were also examined by Western blot analysis to exclude the possibility of CD44 expression being an artifact induced by *in vitro* culture. Different levels of CD44 expression were once again observed in these osteosarcoma patient samples, indicating endogenous expression of CD44 in tumor cells. (Fig. 1B). Subsequently, we performed immunohistochemistry to evaluate the significance of CD44 in a large number of osteosarcoma patients. The TMA used in this study consisted of 14 biopsy samples (considered as primary samples without pre-operative chemotherapy), 56 primary samples, 35 metastatic samples and nine recurrent samples. In total, 114 paraffin blocks were selected to build the TMA. Consistent with the tissue western blot result (Fig. 1B), osteosarcoma specimens presented various degrees of CD44 expression staining on the cell membrane (Fig. 1F). The expression levels of CD44 were significantly higher in metastatic tumors than in primary osteosarcoma tissues (*P* value of metastatic vs. primary <0.0001, Fig. 1C). There was a tendency for increased CD44 expression in recurrent osteosarcoma (Fig. 1C); however, no significant difference was observed between recurrent and primary samples (*P* value of recurrent vs. primary = 0.308). These results may reflect the small number of cases of recurrent specimens in the TMA. There were only six samples applicable for immunostaining analysis due to detachment during the experimental procedures.

### Upregulation of CD44 is correlated with poor chemotherapy response and unfavorable clinical outcome

To investigate the clinical relevance between CD44 expression and osteosarcoma, we analyzed the relation of CD44 expression with osteosarcoma progression and prognosis. Among the 114 tissues selected in this TMA, 77 tissues were treated with chemotherapeutic drugs before undergoing surgery. The proportion of histological necrosis in tumors was carefully evaluated after surgical resection. Previous reports have shown that in the treatment of osteosarcoma, a good response is defined as more than 90% necrosis in the excised tumor, and poor response with the criteria is less than 90% histological necrosis^16^. In the specimens of this TMA, nine patients showed good response, while 47 tissues presented poor response to chemotherapy, and 21 tumor samples were not applicable for determining necrosis (Table 1). The data showed poor response osteosarcoma patients demonstrated a significantly higher level of CD44 expression, in comparison with good response counterparts (*P* value of good response vs. poor response = 0.0484, Fig. 1D, Table 1).

To further prove the clinical significance of CD44 expression in patients with osteosarcoma, the TMA specimen clinical information, including follow-up months, was collected (Table 2 and Table S1). The longest follow-up of a living patient is 255 months. The results showed that shorter overall survival was correlated with stronger expression of CD44. In the Kaplan-Meier survival analysis, CD44 expression scores were subgrouped into two categories: weak CD44 staining with score ranging from 0 to 1+, and strong CD44 staining with score ranging from 2+ to 3+. The survival analysis indicated that the outcome for patients in the CD44 strong-staining group was significantly worse than for those in the CD44 weak-staining group (*P* = 0.0448, Fig. 1E).

### CD44 is a direct target of miR-199a-3p in osteosarcoma

**Figure 2:**
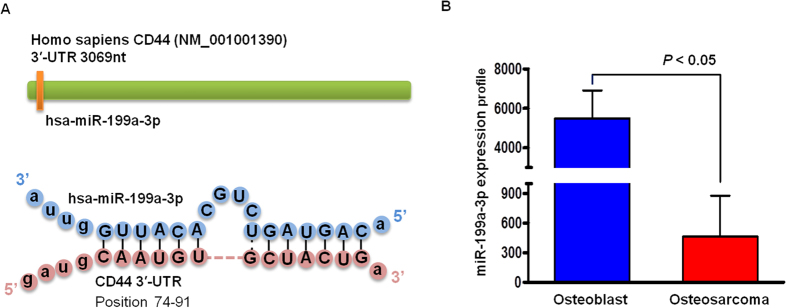
CD44 is a direct target of miR-199a-3p, and the miR-199a-3p expression profile in osteoblast and osteosarcoma cell lines. **A** Alignment of the predicted miR-199a-3p 3′-UTR target sequences of CD44 mRNA. The seed match sequences for miR-199a-3p are indicated by lines. **B** comparison of miR-199a-3p expression levels between in comparison with osteoblasts (HOB-c and NHOst) and osteosarcoma cells (U-2OS and KHOS) based on the miR microarray platform and unsupervised hierarchical clustering analysis.

To identify the specific miR that plays significant roles in cellular motility, adhesion, and invasion in osteosarcoma through targeting CD44, we employed a number of widely used computational analysis databases, including microRNA.org and TargetScan databases, to search for putative binding sites of some miRs in the 3′-UTR of human CD44 gene mRNA. One of the top ranked and highest scoring miRs was miR-199a-3p. Further analysis showed that the alignment of predicted miR-199a-3p target sequences in CD44 mRNA reside at nucleotide 74 to 91 from the start of the CD44 3′-UTR (Fig. 2A). Therefore, miR-199a-3p could be a potential regulator of CD44 expression. Importantly, our previous study has demonstrated that the expression of miR-199a-3p was dramatically decreased in osteosarcoma cell lines (U-2OS and KHOS) in comparison with normal osteoblast (HOB-c and NHOst) cells (Fig. 2B)^15^.

**Figure 3:**
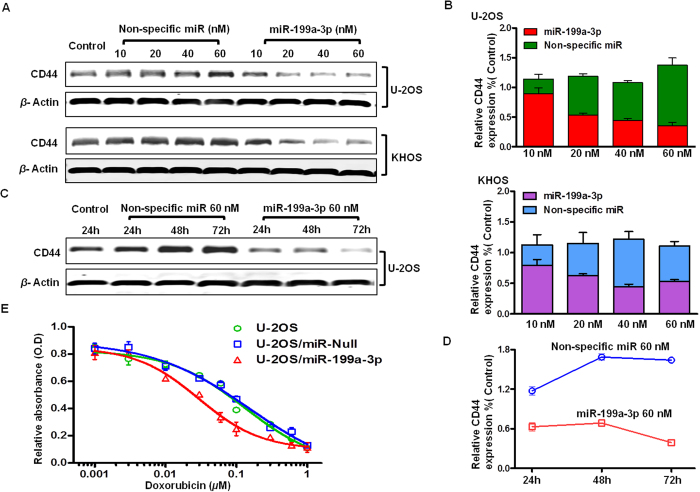
The dose and time dependent effect of miR-199a-3p on knocking down CD44 and increasing the doxorubicin sensitivity of osteosarcoma. **A** and **B** miR-199a-3p knocked down CD44 in osteosarcoma cells as a dose dependent manner. **C** and **D** miR-199a-3p knocked down CD44 in osteosarcoma cells as a time dependent manner. **E** MTT assay showing cell viability of U-2OS, U-2OS/miR-null, and U-2OS/miR-199a-3p after incubating with different concentrations of doxorubicin.

In order to evaluate whether CD44 is a direct target of miR-199a-3p, osteosarcoma cell lines U-2OS and KHOS were transfected with miR-199a-3p mimic, and the effects on CD44 expression were determined. As revealed by Western blot analysis, levels of CD44 were significantly suppressed in the cells treated with miR-199a-3p in a dose-dependent manner (Fig. 3A,B). Specifically, when normalized to control cells, the expression levels of CD44 in cells transfected with 10 nM, 20 nM, and 40 nM miR-199a-3p mimic were decreased to 80.4%, 53.3%, 29.2%, and 18.3% for U-2OS, and 75.5%, 68.7%, 39.2%, and 43.1% for KHOS. In addition, protein from U-2OS dosed with 60 nM miR-199a-3p was collected at different time points to determine the time-dependent knockdown effect of CD44. CD44 expression was decreased by 51.0%, 66.7%, and 75.8% at 24 h, 48 h, and 72 h, respectively. These results suggested that miR-199a-3p repressed CD44 expression in both a dose-dependent and time-dependent manner.

### miR-199a-3p transfection increased drug sensitivity by knocking down CD44 in osteosarcoma

Expression of CD44 has been found to be associated with chemoresistance and recurrence during cancer metastasis and progression. Dysregulation of miRs has also been associated with drug resistance in different tumors. To examine whether repression of CD44 levels by miR-199a-3p would increase drug sensitivity of osteosarcoma cells, the MTT assay was performed on our previously established cell lines U-2OS/null and U-2OS/miR-199a-3p after incubating with doxorubicin. Transfection of miR-199a-3p significantly restored the sensitivity to chemotherapeutic drug doxorubicin in U-2OS. Considering the clinical data we obtained that osteosarcoma patients who responded poorly to chemotherapy presented stronger CD44 levels, it is hypothesized that miR-199a-3p may attenuate drug resistance through inhibition of CD44 expression.

## Discussion

Our current study indicated that upregulation of CD44 is correlated with aggressive behaviors during osteosarcoma progression, including metastasis, recurrence, poor response to chemotherapy, as well as unfavorable prognosis. We demonstrated that CD44 is a direct target of miR-199a-3p in osteosarcoma. miR-199a-3p may mediate the expression level of CD44 and thus impact the drug sensitivity of osteosarcoma cells. These data suggest that the CD44-miR-199a-3p axis may be involved in the development of osteosarcoma.

CD44 functions in cell-matrix interactions, cell motility, matrix degradation, as well as proliferation^7^. CD44 expression is up-regulated in high-grade human breast tumors, and causally contributes to the epithelial-mesenchymal transition and breast cancer progression^17^. CD44 also induces attributes of cells that have undergone an epithelial-mesenchymal transition, leading to tumor metastasis, and resulting in a worse prognosis for colon cancer^18^. CD44 is highly overexpressed in metastatic ovarian cancer cells than in cells isolated from primary tumors^19^,^20^. Moreover, knocking down CD44 by its specific esiRNA dramatically suppressed the migratory potentials and invasive ability of ovarian cancer cell lines in our recent work^21^. The CD44-HA interaction was shown to enhance lung metastasis in osteosarcoma cells^10^. Based on previous *in vivo* investigations, mice with disruption of the CD44 gene showed ablated metastasis formation in osteosarcoma^9^. Additionally, upregulation of CD44 accelerated lung metastasis in mouse osteosarcoma models^11^. In line with these reports, we again confirmed that overexpression of CD44 protein was exhibited in aggressive osteosarcoma cell lines compared with normal osteoblasts. Moreover, we also showed that human osteosarcoma tissues across large sample numbers deriving from late stages (metastatic and recurrent stages) have higher CD44 protein expression than tumor samples in the primary stage. Therefore, CD44 may indeed be a critical modulator in the metastatic and recurrent progression of osteosarcoma.

Currently, one of the most reproducible prognostic indicators for osteosarcoma patients is histological necrosis of tumor tissues to preoperative chemotherapy^22^. A low percentage of necrosis in tumors denotes poor response to chemotherapy and will frequently result in failure of treatment^23^. Our results showed that poor response osteosarcoma patients with less necrotic tumor demonstrated a significantly higher level of CD44 expression, in comparison with good response patients with a high percentage of necrotic tumors. As we reported recently, stable knocking down CD44 by lentivirus-based CD44 shRNA could increase sensitivity to paclitaxel in ovarian cancer cells^21^. CD44 has been reported to be as an anti-apoptotic factor through upregulation of Bcl-xL in doxorubicin resistant breast cancer^24^. Overexpression of CD44 has also been shown to potentiate resistance to etoposide by alteration of caspase 9, caspase 3, Bcl-xl, Bak, pRB, and phosphorylation of AKT in colon cancer^25^. Osteosarcoma is still characterized by the development of drug resistance to chemotherapy in late stages^26^. Although the molecular mechanisms elucidating osteosarcoma chemoresistance remains unknown, CD44 may play a pivotal part in chemoresistance on the basis of our analysis in clinical osteosarcoma tissues.

Association of CD44 expression and clinical prognosis of patients has been studied in diverse malignant tumors, such as colon and breast cancer. Our and others’ previous work have demonstrated that ovarian cancer patients with CD44 positive tumors have a significantly shorter disease free survival than patients with CD44 negative tumors^21^,^27^,^28^. However, the prognostic value of CD44 in osteosarcoma has remained largely unknown. Although overexpression of CD44 was indicated as an important prognostic parameter in 39 patients with osteosarcoma in a previous study, another group showed that CD44 immunoexpression could not predict the prognosis for 34 osteosarcoma patients^17^,^29^. Significant correlation between CD44 expression and overall survival was not observed within 53 osteosarcoma subjects in a recent report^10^. This controversial data may be largely due to the limited number of cases or incomplete clinical information. Notably, the TMA utilized in our current study consisted of a large number of osteosarcoma tissues and more complete clinical information. The TMA analysis revealed a significant correlation between stronger CD44 expression in surgical osteosarcoma specimens with shorter overall survival.

**Figure 4:**
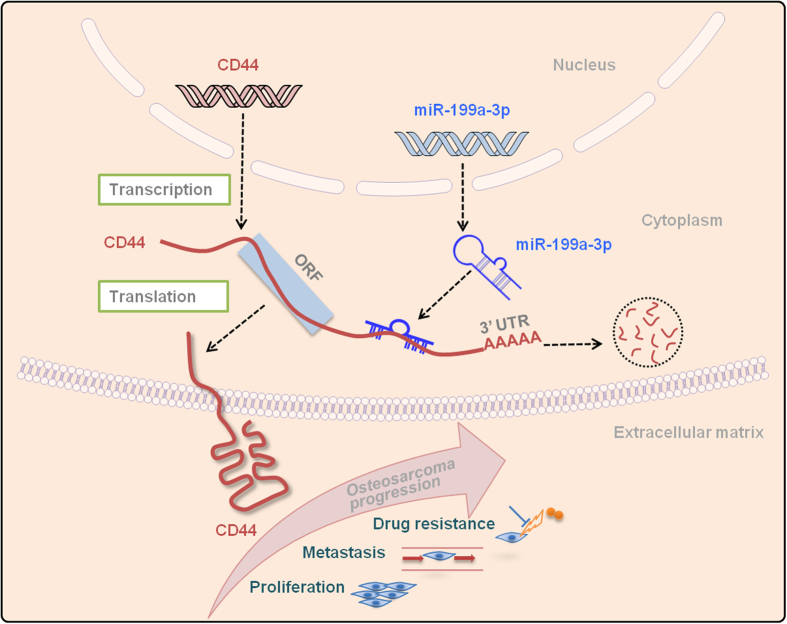
Schematic representation showing CD44-miR-199a-3p axis mediated aggressive behaviors in osteosarcoma. The miR-199a-3p can bind the site within the 3′-UTR of CD44 mRNA, which leads to degradation of the bound RNA. Upregulation of miR-199a-3p in osteosarcoma reduces the expression of CD44 mRNA and leads to decreased translation of the functional CD44 protein. On the other hand, overexpression of CD44 oncoprotein plays critical roles in promoting proliferation, metastasis, and drug resistance during osteosarcoma progression.

Investigations on miRs have unveiled a novel mechanism of post-transcriptional modification of some specific genes that are greatly dysregulated in malignant cells^30^. Based on the TMA results, we then explored the regulation mechanism of CD44 in osteosarcoma. CD44 is mediated by some miRs via binding to its 3′-UTR region in diverse human diseases; for instance, miR-34a in prostate cancer^13^, miR-328 in gastrointestinal cancer^14^, and miR-373 and miR-520c in breast cancer^31^. Recent studies have demonstrated that miRs might influence the progression of osteosarcoma via pathways involving CD44, such as miR-140 and miR-215^32^,^33^. The identification of cancer-specific miRs and their targets is important for uncovering their roles in tumor carcinogenesis and development, and may be pivotal for the discovery of novel therapeutic targets. miR-199a-3p is proven to be a significantly decreased miR in different tumors by our and others’ works, including in osteosarcoma^15^,^34^,^35^. Moreover, transfection of miR-199a-3p was reported to reduce the cellular proliferation rate and influence doxorubicin sensitivity in liver cancer cells, and decrease osteosarcoma cell growth and migration^15^,^36^. As a tumor suppressor miR, miR-199a-3p was demonstrated to down-regulate Met, mTOR and AXL oncogenes^15^,^34^,^37^. Here, we found that CD44 is also a direct target of miR-199a-3p in osteosarcoma. Our findings are consistent with recent reports showing that miR-199a-3p can mediate CD44 expression in hepatocelluar carcinoma and ovarian cancer^38^,^39^. Moreover, miR-199a-3p inhibits the expression of CD44 in osteosarcoma both in a time and dose-dependent manner. In accordance with a former study, down-regulation of CD44 by miR-199a-3p remarkably increased the chemosensitivity to cisplatin, paclitaxel, and adriamycin in ovarian cancer cells^39^. It is assumed that the enhancement of doxorubicin sensitivity in our study may be induced by loss of function of CD44 when miR-199a-3p concentration is enriched inside osteosarcoma cells. It is worth mentioning here that significantly decreased osteosarcoma cell proliferation and migration has been found by restoration of miR-199a-3p^15^. Taking the aforementioned functions of CD44 and miR-199a-3p contributed by our and others’ endeavors into account, we hypothesized that the CD44-miR-199a-3p axis plays an important role in the development of metastasis, recurrence, and drug resistance of osteosarcoma (Fig. 4). Aberrantly expressed miR-199a-3p in osteosarcoma leads to the degradation of CD44 mRNA and further generates decreased translation of CD44 protein. Overexpression of CD44 functions in promoting proliferation, metastasis, and drug resistance during osteosarcoma progression.

In conclusion, findings presented in this study refined our insights into the contributions of CD44 to tumorigenesis, metastasis, and chemoresistance. Our results also underscore the pivotal role of the CD44/miR-199a-3p interaction in determining malignancy in osteosarcoma. Therefore, CD44 may become a therapeutic target in osteosarcoma.

## Methods

### Human osteosarcoma tissues microarray (TMA) construction

The formalin-fixed, paraffin-embedded tumor specimens from 114 osteosarcoma tissues were selected for construction of TMA in the Tissue Microarray Core at the Dana-Farber/Harvard Cancer Center. Haematoxylin and eosin-stained slides from each tissue block were read by a pathologist, together with pathology reports to obtain representative triplicate 0.5-mm-diameter core biopsies. Clinical information of all the subjects was collected and managed from the archives, including age, gender, tumor location, tumor stage (primary/metastasis/recurrent), whether the patient received pre-operative chemotherapy or not, the percentage of necrosis of tumor assessed by a senior consultant pathologist if the patient received chemotherapy, the disease status, as well as the follow-up months (Table 1, Table 2 and Supplementary data Table S1). Institutional Review Board (IRB) approval was obtained to collect all osteosarcoma samples from the Partners Human Research Office. Written informed consent was obtained from all patients whose specimens and clinical information were used for this research study. All experimental protocols were approved by Massachusetts General Hospital (IRB protocol number: 2007P-002464). The methods described in this study were carried out in accordance with the approved guidelines.

### Human osteoblast and osteosarcoma cell lines culture

Human osteoblast cell line HOB-c was purchased from PromoCell GmbH (Heidelberg, Germany), osteoblast cell line NHOst was purchased from Lonza Wallkersville Inc. (Walkersville, MD). Osteoblast cell lines were cultured in osteoblast growth medium (PomoCell) with supplement Mix. The human osteosarcoma cell line KHOS was kindly provided by Dr. Efstathios Gonos (Institute of Biological Research & Biotechnology, Athens, Greece). The human osteosarcoma cell line U-2OS was obtained from the American Type Culture Collection (Rockville, Maryland, USA). Osteosarcoma cell lines were cultured in RPMI 1640 (Life Technologies, Grand Island, NY) supplemented with 10% fetal bovine serum (FBS) and 1% penicillin/streptomycin (Life Technologies, Carlsbad, CA). All cells were incubated in a humidified atmosphere composed of 5% CO_2_ and 95% air.

### Immunohistochemistry

The expression levels of CD44 were evaluated following the Immunohistochemistry Protocol (Cell Signaling Technology, Beverly, MA). In brief, 5-μm paraffin tissue section slides were baked for 1 hour at 60 °C, deparaffinized in xylene three times for 10 minutes each, and then transferred through graded ethanol (100%, 95%) for rehydration. After antigen retrieval, 3% hydrogen peroxide was applied to quench the endogenous peroxidase for 10 minutes. Following protein blocking for 1 hour at room temperature, the slide was incubated with primary antibody (dilution 1:50) at 4 °C overnight in a humidified chamber. Subsequently, bound antibody on the array was detected by SignalStain^®^ Boost Detection Reagent (Cell Signaling Technology) and SignalStain^®^ DAB (Cell Signaling Technology). To obtain better images and long-term preservation, the section was counterstained with hematoxylin QS (Vector Laboratories) and mounted with VectaMount AQ (Vector Laboratories), respectively. The immunostaining intensity of CD44 was assessed independently by two scientists who had no knowledge of the clinical information, as follows: 0, no staining; 1+, weak staining; 2+, moderate staining; and 3+, intense staining. The negative and positive staining controls are shown in Supplementary data Figure S1).

### Western blot

Protein lysates of the cells and tissues were extracted with 1 × RIPA lysis buffer (Upstate Biotechnology, Charlottesville, VA) supplemented with complete protease inhibitor cocktail tablets (Roche Applied Science, IN, USA). The protein concentrations were determined by Protein Assay Reagents (Bio-Rad, Hercules, USA). Equal amounts of protein were separated by NuPAGE^®^ 4–12% Bis-Tris Gel (Life Technologies), transferred onto nitrocellulose membrane (Bio-Rad), and incubated with specific primary antibodies CD44 (Cell Signaling, dilution 1:1000) and *β*-Actin (Sigma-Aldrich, dilution 1:2000) at 4 °C overnight. The membranes were further probed with respective secondary antibodies (LI-COR Bioscences, Lincoln, NE), and scanned by Odyssey^®^ CLx equipment (LI-COR Bioscences) to detect the bands and the density.

### miR-199a-3p mimic/precursor transfection

Transfection of synthetic miR-199a-3p mimic (Ambion® mirVanaTM miR-199a-3p mimics, TX) into KHOS and U-2OS cells was performed with Lipofectamine^®^ RNAiMAX Reagent (Invitrogen) according to the manufacturer’s instructions. A non-specific miR mimic (Ambion^®^ mirVanaTM miRNA mimics Negative Control) was used as a negative control. Forty-eight or 72 hours post-transfection, the proteins were extracted and stored at −80 °C for further study. Transfection of expression vector-based miR-199a-3p precursor and miRNASelect™ pEP-miR-Null (Cell Biolab, Inc, San Diego, CA) into U-2OS, and selection of stable clones was performed as previously described^15^.

### Chemotherapy drug sensitivity analysis

The cytotoxicity of anticancer drug doxorubicin to osteosarcoma cells were assessed by MTT (3(4,5-dimethylthiazol-2-yl)-2,5-diphenyl tetrazolium bromide) assay. U-2OS, U-2OS/miR-null, and U-2OS/miR-199a-3p cells were seeded into 96-well microplates at a density of 1 × 10^3^ cells per well and incubated with a series of concentrations of doxorubicin, which were supplied by the pharmacy at the Massachusetts General Hospital. After 5-day exposure to doxorubicin, 20 μL MTT (Sigma-Aldrich) was dosed and then incubated for another 4 hours at 37 °C and 5% CO_2_ atmosphere. The resulting intracellular formazan crystals was solubilized in acid-isopropanol. Finally, the absorbances were assessed on a SpectraMax Microplate^®^ Spectrophotometer (Molecular Devices LLC, Sunnyvale, CA) at 490 nm. The MTT assay was conducted in triplicate.

### Statistical analysis

The data were analyzed using Prism 5.0 software (Graph Pad Software Inc., San Diego, CA), and expressed as mean ± SEM. Statistical significance was assessed using independent two-tailed Student t-tests for independent data. Overall survival was analyzed using Kaplan-Meier survival curves with Log-rank (Mantel-Cox) Test for significance. Differences of *P* < 0.05 were considered significant for all statistical tests.

## Additional Information

**How to cite this article**: Gao, Y. *et al.* CD44 is a direct target of miR-199a-3p and contributes to aggressive progression in osteosarcoma. *Sci. Rep.* **5**, 11365; doi: 10.1038/srep11365 (2015).

## Supplementary Material

Supplementary Information

## Figures and Tables

**Table 1:** Histological response to pre-operative chemotherapy and CD44 immunostaining.

	Good response* **(% pre-operative chemotherapy)**	Poor response* **(% pre-operative chemotherapy)**	N/A* **(% pre-operative chemotherapy)**
**CD44 High staining**	2 (22.2%)	25 (53.2%)	18 (85.7%)
**CD44 Weak staining**	7(77.8%)	22 (46.8%)	3 (14.3%)
**Total**	9	47	21

^*^Good response: ≥ 90% necrosis; Poor response: <90% necrosis; N/A: not applicable.

**Table 2:** Characteristics of the study osteosarcoma patients.

**Characteristic**	
**Follow up period Average (range), months**	80.96 (0–255)
**Age at diagnosis Average (range), years**	30.55 (6–77)
** >40**	28
** <=40**	86
**Gender**	
** Male**	73
** Female**	41
**Treatment status**	
** Pre-operative chemotherapy (%)**	77 (67.5%)
** No pre-operative chemotherapy (%)**	37 (32.5%)
**Stages**	
** Primary**	70
** Metastatic**	35
Recurrent*	9

^*^There were only 6 samples applicable for immunostaining analysis due to the detachment during experimental procedures.
