# Supplementary material for: CD44 is a direct target of miR-199a-3p and contributes to aggressive progression in osteosarcoma
Source: Sci Rep. 2015 Jun 16;5:11365. doi: 10.1038/srep11365 (PMC4468826; doi:10.1038/srep11365)
Supplement: Supplementary Information [file srep11365-s1.pdf]

Title: CD44 is a direct target of miR-199a-3p and contributes to aggressive progression in osteosarcoma

Yan Gao[1,2], Yong Feng[1], Jacson K. Shen[1], Min Lin[1], Edwin Choy[1], Gregory M. Cote[1], David C. Harmon[1], Henry J. Mankin[1], Francis J. Hornicek[1], Zhenfeng Duan[1,*]

1 Sarcoma Biology Laboratory, Center for Sarcoma and Connective Tissue Oncology, Massachusetts General Hospital and Harvard Medical School, Boston, MA 02114

2 Department of Clinical Laboratory, The Third Affiliated Hospital of Zhengzhou University, Zhengzhou 450052, Henan Province, China

* Corresponding author: Zhenfeng Duan, Sarcoma Biology Laboratory, Center for Sarcoma and Connective Tissue Oncology, Massachusetts General Hospital, 100 Blossom St, Jackson 1115, Boston, MA 02114, USA. Phone: 617-724-3144; Fax: 617-726-3883.

E-mail address: zduan@mgh.harvard.edu

Table S1. Tumor locations of the study osteosarcoma patients

Stages	Tumor location			
**Primary (70 tissues)**	Anterior mediastinum	1	Right clavicle	1
	Chest wall	1	Right distal femur	16
	Left distal femur	13	Right distal radius	1
	Left foot	2	Right hemipelvis	1
	Left ischium	2	Right humerus	1
	Left mid-humerus	1	Right proximal femur	2
	Left pelvis	1	Right proximal fibula	1
	Left proximal femur	1	Right proximal humerus	3
	Left proximal tibia	5	Right proximal tibia	5
	Left superior pubic ramus	1	Right pubic ramus and acetabulu	1
	Left tibia	4	Right scapula	2
	Maxilla	1	Right thigh	1
	Right acetabulum	1	T10	1
			**Origin location**	
**Metastatic (35 tissues)**	Brain	1	Left distal femur	5
	Chest wall	2	Left foot origin	1
	Heart	1	Left ischium origin	1
	Left hemipelvis	1	Left proximal humerus origin	1
	Lung	23	Right clavicle origin	2
	pelvis and abdomen	1	Right distal femur origin	7
	Right ilium	1	Right proximal femur origin	4
	Right pelvis	1	Right proximal humerus origin	3
	Right proximal humerus	1	Left tibia origin	3
	Right proximal posterior thig	1	Left chest wall	1
	Spine	1	Left proximal femur origin	1
	T2	1	Right ilium Left femur origin	1
			Right thigh origin	1
			Right tibia origin	3
			T2	1
			**Origin location**	
**Recurrent (9 tissues)**	Left femur	1	Right chest wall	1
	Left pelvis	1	Right proximal tibia origin	1
	Right acetabulum	1	Unknown	7
	Right bicep	1		
	Right chest wall	1		
	Right distal femur	2		
	Right proximal radius	1		
	Right shoulder	1		

Figure S1. Negative and positive controls of immunohistochemistry

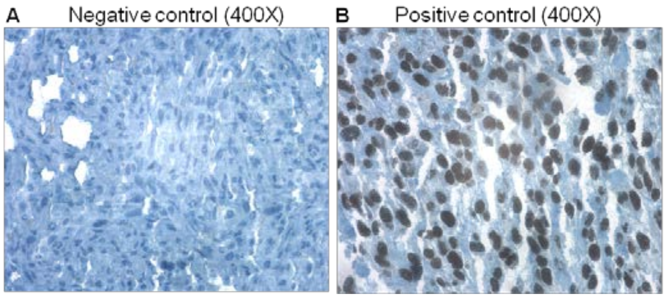

A, osteosarcoma tissue incubated with antibody diluents only, without primary antibody (CD44 or enhancer of zeste homolog 2, EZH2) included was used as the negative control of immunohistochemistry assay. B, immunohistochemistry staining of EZH2 in osteosarcoma was used as the positive control, which is located in nucleus.
